# Exercise for Osteoarthritis: A Literature Review of Pathology and Mechanism

**DOI:** 10.3389/fnagi.2022.854026

**Published:** 2022-05-03

**Authors:** Hui Kong, Xue-Qiang Wang, Xin-An Zhang

**Affiliations:** ^1^College of Kinesiology, Shenyang Sport University, Shenyang, China; ^2^Department of Sport Rehabilitation, Shanghai University of Sport, Shanghai, China; ^3^Department of Rehabilitation Medicine, Shanghai Shangti Orthopedic Hospital, Shanghai, China

**Keywords:** osteoarthritis, exercise, pathology, mechanism, therapy

## Abstract

Osteoarthritis (OA) has a very high incidence worldwide and has become a very common joint disease in the elderly. Currently, the treatment methods for OA include surgery, drug therapy, and exercise therapy. In recent years, the treatment of certain diseases by exercise has received increasing research and attention. Proper exercise can improve the physiological function of various organs of the body. At present, the treatment of OA is usually symptomatic. Limited methods are available for the treatment of OA according to its pathogenesis, and effective intervention has not been developed to slow down the progress of OA from the molecular level. Only by clarifying the mechanism of exercise treatment of OA and the influence of different exercise intensities on OA patients can we choose the appropriate exercise prescription to prevent and treat OA. This review mainly expounds the mechanism that exercise alleviates the pathological changes of OA by affecting the degradation of the ECM, apoptosis, inflammatory response, autophagy, and changes of ncRNA, and summarizes the effects of different exercise types on OA patients. Finally, it is found that different exercise types, exercise intensity, exercise time and exercise frequency have different effects on OA patients. At the same time, suitable exercise prescriptions are recommended for OA patients.

## Introduction

Osteoarthritis (OA) has a very high incidence worldwide, and it is strongly associated with age (van Saase et al., 1989). Most of the patients with OA are aged over 60. With the aging of the population, the disease has become a common joint disease today. Heredity, hormones, diet, obesity, smoking, and drinking can lead to OA (Felson et al., 1997). Its early symptoms mainly include pain and joint stiffness, and later secondary changes such as muscle atrophy and joint contracture occur (Bennell et al., 2012). Therefore, we advocate early intervention for patients with OA to prevent further deterioration of the disease. However, the disease treatment still encounters many shortcomings, and further research and exploration are needed. Currently, the treatment methods for OA include surgery, drug therapy, and exercise therapy (Spahn et al., 2013). In recent years, the treatment of certain diseases by exercise has received increasing research and attention. Exercise is an economical and effective treatment (Ettinger et al., 1997). Proper exercise can improve the physiological function of various organs of the body and improve the overall morphology of the body (Benedetti et al., 2018). In addition, exercise can also relieve pain (Belavy et al., 2021; Zheng et al., 2021; Peng et al., 2022; Wu et al., 2022). For OA patients, ladder treatment is generally adopted, starting with basic treatment. If it is ineffective, drug therapy or surgery can be used, and exercise therapy is one of the basic treatment methods. The effects of different exercise types on OA have been widely studied.

At present, the treatment of OA is usually symptomatic. Limited methods are available for the treatment of OA according to its pathogenesis. Exercise can alleviate OA at a molecular level. Only by clarifying the mechanism of exercise treatment of OA and the influence of different exercise intensities on normal joints and OA patients can we choose the appropriate exercise prescription to prevent and treat OA. This review mainly expounds the mechanism that exercise alleviates the pathological changes of OA by affecting the degradation of the ECM, apoptosis, inflammatory response, autophagy, and changes of ncRNA, and summarizes the effects of different exercise types on OA patients. Finally, it is found that different exercise types, exercise intensity, exercise time and exercise frequency have different effects on OA patients. At the same time, suitable exercise prescriptions are recommended for OA patients.

## Pathological Change Mechanism of Osteoarthritis

For OA patients, the integrity of the entire joint tissue is damaged, including articular cartilage, subchondral bone, and synovial membrane (Loeser et al., 2012). Articular cartilage is mainly composed of the extracellular matrix (ECM), consisting of water, collagen, proteoglycans, mucopolysaccharides, type II collagen, and chondrocytes (Luo et al., 2017). With the presence of prolonged, excessive mechanical stimulation of the body, articular cartilage will suffer. In the early stage of articular cartilage injury, the concentration of growth factors in the ECM increases as chondrocytes gather in the damaged area, resulting in transient cell proliferation and ECM synthesis (Suri and Walsh, 2012). As the damage worsens, the blood supply to the articular cartilage worsens to the point that the cartilage does not have adequate access to nutrients. This condition results in cartilage cell apoptosis, ECM synthesis, and the disappearance of the articular cartilage degeneration (Eyre, 2004). The two bones constantly rub, resulting in joint pain, swelling, and function limitation. Its continuous development causes OA (Rim et al., 2020). Subchondral bone includes subchondral cancellous bone and cortical plates. X-ray and Magnetic Resonance Imaging (MRI) diagnosis can reveal the abnormal remodeling of subchondral bone and a series of changes in bonemorphology, such as bone spurs, osteophytes, and wear in OA patients (Braun and Gold, 2012). This sequence of changes may be caused by an imbalance in the production and destruction of osteoblasts and osteoclasts (Burr and Gallant, 2012). This morphological change of subchondral bone may also cause damage to the articular cartilage overlying it. Patients with OA have an exceptionally high probability of suffering from synovitis, and this condition is related to the pain and function of the knee joint during OA development (Sowers et al., 2011). The synovial membrane is the connective tissue membrane covered on the inner surface of the joint capsule, which can produce synovial fluid to reduce joint friction and the loss of cartilage and ensure the metabolism and nutrition supply of joint cartilage (Pap et al., 2020). Synovial cells mainly include synovial macrophages, fibroblast-like synovial cells, and mesenchymal stem cells. The macrophages can engulf the damaged tissue. When synovitis occurs in the body, the macrophages decompose to produce inflammatory factors, and the generation and degradation of cartilage matrix are in dynamic balance under normal conditions. Inflammatory mediators can destroy this balance, decompose chondrocytes, and aggravate synovitis (Da et al., 2007). Fibroblast-like synovial cells can produce hyaluronic acid, which is an essential component of joint synovial fluid. During OA progression, the content of hyaluronic acid is remarkably reduced, resulting in the reduction of joint function and damage to the integrity of the joint surface (Scanzello and Goldring, 2012).

**FIGURE 1:**
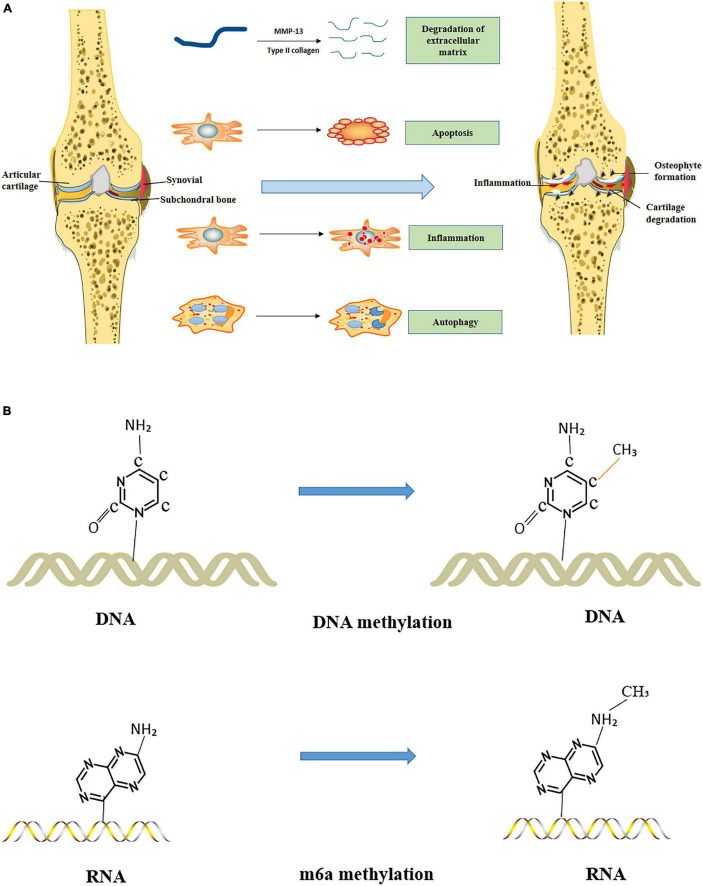
Pathogenesis of Osteoarthritis (OA). **(A)** The degradation of ECM, apoptosis, inflammatory response, and autophagy mechanisms in OA. **(B)** Methylation in OA.

Above all, articular cartilage, synovium, and subchondral bone abnormalities are the main pathological changes of OA (Valdes and Spector, 2010). Considering that articular cartilage receives nutrients through synovial fluid, articular cartilage in patients with synovitis may undergo pathological changes. Moreover, the increase of macrophages in patients with synovitis may cause subchondral bones in OA to form osteophytes. Ayral et al. (2005) evaluated the correlation between synovitis and the severity of cartilage structure damage in OA patients through a one-year multicenter longitudinal study involving 422 patients. After arthroscopic and pain scoring, they found that compared with patients with normal synovial membrane, patients with synovitis had more severe articular cartilage lesions. The degree of deterioration after 1 year was statistically different. Yusup et al. (2015) studied 40 patients with knee OA and scored the structures such as joint cartilage, subchondral bone, and synovial membrane by using 3.0-T MRI and found that synovitis mainly occurred in the early and late stage of OA patients. The destruction of joint cartilage and a series of changes in subchondral bone could induce synovitis, and a strong correlation was observed among the three parameters. Some studies (Norrdin et al., 1998) use horse as a model to study the pathological changes of OA, in which the morphological changes of subchondral bone occur before articular cartilage injury. In other studies (Brandt et al., 1991), dog is used as the model to investigate the pathological changes of OA, in which the morphological changes of early subchondral bone coincided with an articular cartilage injury. Generally, the pathological mechanisms of articular cartilage, synovium, and subchondral bone in OA mainly include the degradation of ECM, cell proliferation, cell apoptosis, inflammatory reaction, changes of non-coding RNA, and methylation (Mobasheri et al., 2017) (Figure 1).

### Degradation of Extracellular Matrix

The ECM is a complex network of large molecules such as polysaccharides and proteins around cells. This structure and special cells make up the cartilage (Theocharis et al., 2019). Many diseases are associated with changes in the composition and properties of ECM, such as OA. In healthy articular cartilage, the synthesis and metabolism of ECM should always maintain a dynamic balance to maintain homeostasis. The secretion and operation of some components of ECM are completed by articular cartilage (Rahmati et al., 2017). When the activities of synthesis and catabolism of articular cartilage are imbalanced, the details and homeostasis of ECM are also affected (Guo et al., 2002). Considering that the degradation of ECM leads to the loss of cartilage tissue, ECM also plays a role in maintaining the function of chondrocytes. The continuous degradation of ECM will induce OA.

Based on the summarizing of articles related to the pathogenesis of OA and the function of chondrocytes, Goldring and Goldring (2007) found that the degradation of ECM and decomposition of collagen could lead to the gradual loss of the shape and position of articular cartilage to induce OA. Setayeshmehr et al. (2019) summarized the functions and effects of various ECM scaffolds through summarizing and analyzing the related articles on hybrid and composite scaffolds derived from chondrocyte ECM. They concluded that the degradation of chondrocyte ECM could lead to cartilage degeneration. Malemud (2017) analyzed the role of matrix metalloproteinases in OA. They found that in the ECM of the cartilage, some essential proteins would degrade. Matrix metalloproteinases can promote or inhibit the degradation of these proteins, change the biomechanical properties of tissues, and destroy articular cartilage. In addition, based on the analysis of the action mechanism of ECM and its degrading enzymes in OA, the ECM of cartilage in OA patients can release certain fibronectin, thereby inducing the expression of related proteases and degrading the components of the ECM (Pérez-García et al., 2019).

Kapoor et al. (2011) and Theocharis et al. (2019) also found that the tissue protein would be hydrolyzed gradually during the development of OA. Then, some catabolic mediators will be produced, which will induce the expression of some related cytokines and proteases, and finally lead to ECM degradation. This situation will lead to prolonged OA. Based on the analysis of the changes of some macromolecules in the ECM of early OA patients, the content of proteins related to ECM was changed, such as collagen and cartilage protein. Hence, the degradation of ECM and even the deterioration of OA disease occurred (Lorenzo et al., 2004).

### Apoptosis

Apoptosis is an active gene-determined process that automatically ends life, and it is often called programmed cell death, a basic biological phenomenon of cells (Musumeci et al., 2011). Apoptosis involves endogenous and exogenous pathways, which play an essential role in maintaining the function of various tissues in the body (Elmore, 2007). A correlation has been observed between the degree of cartilage injury and apoptosis, which is a vital mechanism of cartilage injury (Hwang and Kim, 2015). Moreover, the apoptosis of OA chondrocytes is related to cartilage degradation (Kim et al., 2003). When OA occurs, it will produce matrix-degrading enzymes, leading to the degradation of the ECM and the destruction of cell homeostasis. Cell stress induces an oxidation reaction, leading to the apoptosis of chondrocytes and regulating the pathological changes of cartilage tissues (Bauer et al., 2006).

Histologically, Musumeci et al. (2011) studied the articular cartilage of normal individuals and OA patients by staining apoptotic cells. They found that the articular cartilage of OA patients changed in structure. The pro-apoptotic receptor on the cell surface could induce apoptosis through an exogenous pathway in the damaged articular cartilage. Aigner et al. (2004) summarized the incidence, induction mechanism, and morphology of apoptosis in OA patients and found that apoptosis occurred in patients with OA, but the incidence was not high. The body induced apoptosis through specific proteoglycans, signaling molecules, and other pathways to regulate the pathological changes of articular cartilage and accelerate or delay the progression of OA. Based on the synthesis of related articles on OA and apoptosis, some articles indicate that apoptosis induces OA, while other articles indicate that OA induces apoptosis. These two views are disputed, and more reports believe that these conditions affect and promote each other and have a correlation (Zamli and Sharif, 2011). Chondrocytes in the knee joints of patients with OA and the apoptosis of chondrocytes and its relationship with cartilage degradation have been studied. Notably, the findings indicate that first, in some populations, the morphology and function of cartilage are normal (Hashimoto et al., 1998). Still, many apoptotic cells appear, indicating that apoptosis may lead to degenerative changes in articular cartilage, thus inducing OA. Furthermore, the number of apoptotic articular cartilage cells in the OA population increased, and the number of apoptotic cells was related to cartilage degradation. Finally, when all subjects, including the average population and OA patients, were analyzed, a correlation was found between age and apoptosis of chondrocytes. Similarly, rabbit chondrocytes have been studied and compared with mature rabbits. Results show that the cell density of each layer of articular cartilage in rabbits decreased, and the expression level of pro-apoptotic genes increased. These studies further proved the correlation between age and chondrocyte apoptosis at the animal level and the phenomenon of apoptosis that existed in the early stage of OA (Todd Allen et al., 2004). In another study involving horses, a positive correlation was found between the severity of chondrocyte injury and apoptosis. The expression level of pro-apoptotic factors is higher in horses that often suffer from OA. Apoptosis participates in the pathogenesis of OA. Apoptosis has been observed on human femoral head cartilage (Thomas et al., 2011). In comparison with the average population, the expression of receptors and the degree of apoptosis in OA patients’ articular cartilage was much higher. In the late stages of OA, the degree of apoptosis become even worse (Héraud et al., 2000).

### Inflammatory Reaction

The inflammatory reaction is a basic pathological process mainly involving a defensive response when the body is stimulated. Inflammation is involved in the pathological process of many diseases, such as cancer (Gianni et al., 2016), tendon or ligament injury (Gracey et al., 2020). As early as the middle of the 19th century, studies pointed out that inflammation was closely related to OA. Articular chondrocytes and synovial cells all expressed inflammatory mediators (Liu-Bryan, 2013). Inflammatory factors such as IL-1β and TNFα participate in the inflammatory response in articular chondrocytes and synovial cells (Malfait, 2016). The cells mainly involved in the inflammatory response are macrophages and monocytes. The degree of inflammation is related to the degree of joint dysfunction and inflammatory factors (Scanzello et al., 2011). In the early stage of OA, the expression of monocytes and inflammatory mediators increased, whereas with the continuous development of OA, the expression of monocytes and inflammatory mediators was gradually decreased (Benito et al., 2005).

Rosshirt et al. (2019) used the knee joints of 55 OA patients as the research object, described the activation state of T cells, and found that a large proportion of T cells were activated to participate in OA inflammation, which mainly affected the joint itself the most. Inflammation can promote catabolism and participate in the pathogenesis of the disease. van den Bosch (2019) found a large number of inflammatory mediators in the tissues of patients with OA. These inflammatory mediators can induce the generation of degrading enzymes in articular cartilage, leading to the degradation of the ECM and the destruction of cartilage tissue. Lieberthal et al. (2015) established a post-traumatic OA model and observed the relationship between inflammation and OA progression. They found that an inflammatory reaction occurred in the early stage of OA. In patients with, multiple inflammatory pathways were activated and produced multiple inflammatory mediators such as cytokines and chemokines, which played an essential role in the pathogenesis of OA (Scanzello, 2017a). The genomic expression profiles of OA patients have been collected and subjected to meta-analysis, and the results show that MAP kinase, NF-κB activation, and oxidative phosphorylation can induce inflammatory signals and participate in the pathological change mechanism of OA (Li et al., 2014). Macrophages are immune cells that play an essential role in the inflammatory response (Bondeson et al., 2010). Woodell-May and Sommerfeld (2020) explored the role of immune cells such as macrophages in the synovial membrane of patients with OA. They found that in OA, macrophages could release oxygen free radicals, proteases, and inflammatory factors, thus affecting the microenvironment of inflammation. In the wound healing stage, macrophages also release IL-1, IL-6, and other inflammatory factors to regulate the inflammatory response. By stimulating protease, inflammation can cause cartilage degeneration and eventually induce OA. Based on the relationship between mechanical injury and OA, mechanical damage can lead to mechanical inflammation, which activates NF-kB and inflammatory mitogen-activated protein kinase, thereby causing a series of functional problems of the join (Vincent, 2019). If the rash persists for a long time, it will affect the repair of cartilage tissue. Based on the summary of articles related to inflammation in OA (Goldring and Otero, 2011), when some risk factors inducing OA appear, the expression of pro-inflammatory factors and various related enzymes are upregulated in articular cartilage and synovial tissue through specific signaling pathways. Inflammatory elements, which are essential for cartilage damage and repair, can change adjacent joint tissues and form a vicious circle (Houard et al., 2013). The knee joint is damaged under severe mechanical stimulation, causing catabolism and stress response of chondrocytes, and finally inducing inflammation, leading to joint pain. Inflammation is one of the important mechanisms that lead to the pathological changes of joint cartilage and synovial membrane (Scanzello, 2017b).

### Autophagy

During autophagy, cells degrade their damaged organelles and macromolecular substances by using lysosomes to regulate autophagy-related genes, which can achieve the renewal of organelles and is essential for cell metabolism (Guo et al., 2021). Autophagy is involved in the occurrence of OA. Through cellular autophagy, the function of damaged articular cartilage can be restored, thereby alleviating the pathological process of OA. Excess ROS will lead to cartilage degradation and inhibit the synthesis of ECM. Kongara and Karantza (2012) showed that autophagy could maintain the typical morphology of cartilage and the dynamic balance of ECM synthesis and metabolism by regulating the body’s ROS.

Barranco (2015) found that the inhibition of the Akt-mTOR signaling pathway in chondrocytes can promote the autophagy of articular chondrocytes and regulate oxidative stress (Xue et al., 2017), thus participating in the development process of OA. If autophagy is activated, the damaged mitochondria and peroxidase bodies are removed, thus inhibiting the production of reactive oxygen species in the body and protecting the articular cartilage from pathological changes. Based on the summary of the roles of autophagy in OA, in the early stage of OA, moderate autophagy contributes to the survival of chondrocytes and is essential for preventing and delaying OA (Duan et al., 2020). Li et al. (2016) found that autophagy was mainly committed in the chondrocytes of patients with OA. By regulating oxidative stress response and apoptosis, the pathological changes of the knee joint can be alleviated, and chondrocytes can be protected from various stimulations. With the growth of age, the incidence of OA increases gradually, and this phenomenon is related to autophagy. Autophagy gradually weakens with age. Therefore, the structure and function of articular cartilage decreases with age, leading to pathological changes in the knee joint (Mathew et al., 2009). Caramés et al. (2010) that in OA animal models, the expression of autophagy regulatory factors is downregulated, and the steady-state of cartilage is damaged, indicating that autophagy can protect articular cartilage and play an essential role in maintaining cartilage steady state. The decrease of autophagy will induce OA. Taking articular cartilage of OA and non-OA patients as the research object, we can find that autophagy regulates the expression of OA-related genes. In comparison with patients without OA, autophagy is expressed in the chondrocytes of OA patients at a higher level, and the manifestation of autophagy markers is also upregulated (Sasaki et al., 2012). Similarly, Chang et al. (2013) took human chondrocytes as the research object and found that many autophagy-related proteins are expressed in the articular cartilage of patients with OA. In the pathogenesis of OA, autophagy plays a role in protecting chondrocytes and promoting metabolism. However, excessive autophagy causes a large number of chondrocyte deaths.

### Changes in Non-coding RNA

Non-coding RNA (ncRNA) is an RNA that does not code for protein, mainly including miRNA, lncRNA, and circRNA. These RNAs can exert biological functions at the RNA level without being translated into proteins. ncRNA plays an essential role in the pathological process of many diseases, such as cardiovascular disease (Jusic and Devaux, 2020), cancer (Karreth and Pandolfi, 2013), diabetes (Beltrami et al., 2015). ncRNA also plays a vital role in the process of OA. It can promote or inhibit cartilage formation by regulating the degradation of cell-matrix, cell proliferation, apoptosis, and inflammatory response, thereby inducing or treating OA (Hong and Reddi, 2012).

Changes in the expression levels of many miRNAs can induce pathological changes in OA. MiR-204/-211 and miR-29b-3p are common miRNAs, which are differentially expressed in patients with OA. Huang et al. (2019) established an OA mouse model and found that miR-204/-211 was missing, and Runx2 was increased. Mesenchymal progenitor cells proliferated abnormally at the same time by Micro-CT and histological determination. Akt signal North is activated, thus inducing the dysfunction of various components in the joint and finally leading to OA. Therefore, miR-204/-211 can maintain intra-articular homeostasis and ensure that articular chondrocytes and synovial cells function usually. Chen et al. (2017) used rat chondrocytes as the research object. Through luciferase reporter gene detection, they found that miR-29b-3p in patients with OA was upregulated, and miR-29b-3p ultimately led to the degeneration of articular cartilage tissue by targeting and inhibiting the expression of rat GRN mRNA. In addition, microarray technology was used to detect the expression of miRNA in chondrocytes. They found that miRNA-140, miRNA-455, miR-146a, miR-155, and miR125b differed in OA patients and the average population, thus inducing pathological changes of articular cartilage, synovial membrane, and subchondral bone (Swingler et al., 2012). lncRNA also plays a vital role in OA. Throughout the cartilage development, different lncRNA is regulated, and the regular expression of lncRNA can prevent cartilage differentiation disorders. During cartilage degeneration, other lncRNA plays various roles (Zhu et al., 2019). Ye et al. (2018) studied the articular cartilage of patients with OA. They found that the expression of ZFAS1 in OA chondrocytes was downregulated, and ZFAS1 might reduce the activity of chondrocytes and induce pathological changes of articular cartilage tissues by targeting the Wnt3a signaling pathway. Similarly, the role of circa in OA has been widely studied. Cyclic RNA plays a multi-faceted regulatory role in the progression of OA (Zhang W. et al., 2021). Chen et al. (2020) found that in OA tissues, the expression levels of circRNA-UBE2G1 and HIF-1a were significantly increased, while the expression of miR-373 was downregulated. Functional testing showed that circRNA-UBE2G1 could bind to miR-373, thereby inhibiting the interaction of miR-373 and HIF-1a, damaging the chondrocytes, and leading to a series of pathological changes. In conclusion, ncRNA is closely related to OA. The differential expression of miRNA, lncRNA and circRNA in healthy people and OA patients can lead to pathological changes in articular cartilage, synovial membrane, and subchondral bone and affect the pathological process of OA.

### Methylation

Methylation is the catalyzed transfer of methyl groups from an active compound to another compound. This process can form various methyl compounds or result in chemical modification of particular proteins or nucleic acids to start methylation products (Urnov, 2002). Methylation mainly includes DNA methylation and m6a methylation (Dai et al., 2021).

DNA methylation is a chemical modification of DNA that alters genetic behavior without altering the DNA sequence. Its primary process is under the action of DNA methyltransferase. DNA methylation can cause changes in chromatin structure, DNA conformation, DNA stability, and the way DNA interacts with the protein, thereby controlling gene expression (Reynard, 2017). When CtBP1 and CtBP2 are overexpressed, the pro-inflammatory factors are increased, while the NLRP3 signaling pathway is activated to induce OA. DNA methylation in the CtBPs promoter reduces the expression level of CtBPs in OA tissues and regulate CtBP-mediated signal transduction, finally participating in the pathogenesis of OA (Sun et al., 2020). In addition, DNA methylation can participate in the pathogenesis of OA by affecting the expression of genes such as matrix metalloproteinase-3, matrix metalloproteinase-9, and type II collagen (Cui and Xu, 2018).

N6- methyladenine (m6a) is one of the most abundant chemical modifications of eukaryotic messenger RNA. m6a modifications mainly include m6a methyltransferase catalysis, m6a demethylase removal, and m6a binding protein recognition (Ma and Ji, 2020). This process is widely involved in regulating various life cycle stages such as mRNA splicing, processing, translation, and degradation. It is related to osteosarcoma, rheumatoid arthritis, osteoporosis, OA, and abnormal physiological functions (Zhang W. et al., 2020). The strange expression of m6a-related gene and protein in OA can trigger the imbalance of m6a methylation, regulate the expression of OA-related genes to participate in the occurrence and development of OA, and is closely related to the poor prognosis of patients (Yang et al., 2021). METTL3 is a methylated gene of m6a. He et al. (2021) studied mice *in vivo* and *in vitro* and induced the OA model with inflammatory stimuli such as TNF-α. The results showed that the expression of METTL3 was decreased in the OA model. Bcl2 is a downstream target gene of METTL3, and the presentation of METTL3 can promote m6A methylation of Bcl2 mRNA, thereby inhibiting chondrocyte apoptosis and autophagy. The detection of synovial tissue in patients with OA revealed that METTL3 could also induce autophagy by inhibiting ATG7 (Chen X. et al., 2021). In addition, METTL3 may participate in OA by regulating the inflammatory response (Sang et al., 2021). In summary, methylation is also an essential mechanism for pathological changes in OA. Furthermore, the epigenetic mechanism plays an essential role in the pathogenesis of OA.

## Mechanism of Exercise Improving Osteoarthritis

**FIGURE 2:**
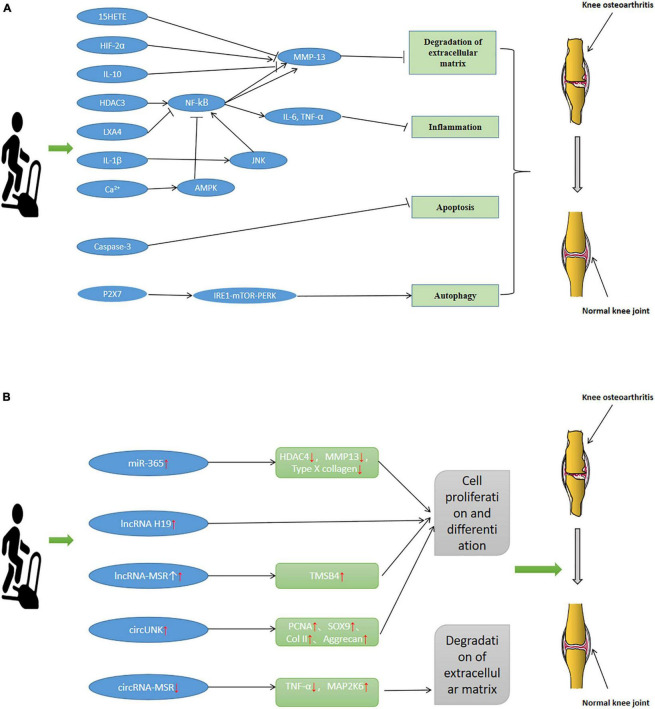
Pathological change mechanism of Osteoarthritis (OA). Exercise relieves the pathological changes of OA by affecting the degradation of the ECM, apoptosis, inflammatory response, autophagy and changes of ncRNA. **(A)** Mechanism of pathological changes of OA through degradation of ECM, apoptosis, inflammatory response, and autophagy. **(B)** Mechanism of pathological changes of OA through ncRNA.

Exercise is good medicine. An increasing number of studies supports that exercise can enhance physical fitness, build up the body, and prevent and treat certain diseases, playing an increasingly important role in people’s lives (Ruegsegger and Booth, 2018). Studies (Pedersen and Saltin, 2015) have summarized the mechanism of exercise in treating 26 different conditions, providing a theoretical basis for treating diseases by exercise. The treatment of OA by exercise has attracted increasing attention and has gradually become an OA research hotspot. In the present paper, the pathological mechanism of exercise in OA treatment was summarized through the induction of relevant literature, as shown in Table 1. Exercise relieves the pathological changes of OA by affecting the degradation of the ECM, apoptosis, inflammatory response, autophagy, and changes of ncRNA. And training is used to treat OA (Figure 2).

**TABLE 1:** Mechanism of exercise in the treatment of Osteoarthritis (OA).

Researchers	Model	Exercise types	Related gene/cytokines/protein	Involved in pathways	Improved organization	Functions	Change
He et al., 2020	OA mice model	Cell stretch	Irisin	Erk	Articular cartilage	Apoptosis	↓
Griffin et al., 2020	OA mice model	Wheel-running exercise	—	—	Subchondral bone	Inflammation	↓
Lu et al., 2021	OA rat model	Treadmill exercise	Maresin-1, MMP-13	PI3K/AKT, NF-κB	Synovial	Inflammation	↓
Griffin et al., 2012	OA mice model	Wheel-running exercise	KC, leptin, IL-1Ra	—	Subchondral bone, Articular cartilage	Inflammation	↓
Cormier et al., 2017	OA rat model	Prior wheel running	—	—	Subchondral bone, Articular cartilage	Inflammation	↓
Chen et al., 2020	OA rat model	Treadmill and wheel exercise	IL-1b, IL-6, TNF-a	JNK/NF-kB	Articular cartilage, synovial	Inflammation	↓
Rios et al., 2019	OA rat model	Aerobic exercise	IL-6, TNF-α	—	Subchondral bone	Inflammation	↓
Li K. et al., 2021	OA mice model	Wheel-running exercise	TLR4, MMP-13	—	Articular cartilage	Degradation of ECM	↓
Yang et al., 2020	OA rat model	Treadmill exercise	TRAIL	TRAIL/NF-κB/NLRP3	Articular cartilage	Apoptosis, Inflammation	↓
Allen et al., 2017	OA rat model	Treadmill exercise	—	—	Subchondral bone	Inflammation	↓
Zhang X. et al., 2019	OA rat model	Treadmill exercise	Lc3B, SQSTM1	—	Articular cartilage	Autophagy	↑
Zhang H. et al., 2019	OA rat model	Treadmill exercise	HDAC3	HDAC3/NF-KappaB	Articular cartilage	Inflammation	↓
Galois et al., 2004	OA rat model	Running training	Hsp70	—	Articular cartilage	Apoptosis	↓
Oka et al., 2019	OA mice model	Treadmill exercise	IL-1β, MMP-13	—	Articular cartilage	Inflammation, Degradation of ECM	↓
Blazek et al., 2016	OA rat model	Treadmill exercise	MMP-13,Type II collagen	—	Articular cartilage	Degradation of ECM	↓
Yang et al., 2019	OA rat model	Treadmill exercise	IL - 1β	AMPK/NF – κB	Articular cartilage	Inflammation	↓
Assis et al., 2016	OA rat model	Aerobic exercise	IL-1β, caspase-3, MMP-13	—	Articular cartilage	Inflammation	↓
Baur et al., 2011	OA mice model	Running exercise	ROS, SOD2	—	Articular cartilage	Autophagy	↓
Assis et al., 2015	OA rat model	Treadmill exercise	muscle-specific ring- finger protein 1, atrogin-1	—	Articular cartilage	Degradation of ECM	↓
Yamaguchi et al., 2013	OA rat model	Running	C2C, CPII	—	Articular cartilage	Degradation of ECM	↓
Assis et al., 2018	OA rat model	An aerobic and an aquatic exercise	IL-10, TGF-β, collagen I and II	—	Articular cartilage	Degradation of ECM	↓
Milares et al., 2016	OA rat model	An aquatic exercise	IL1-β, caspase-3	—	Articular cartilage	Inflammation	↓
Szychlinska et al., 2019	OA rat model	Physical activity	IL-6	—	Articular cartilage	Inflammation	↓
Qian et al., 2014	OA rat model	Passive motion	Proteoglycans, type II collagen fibers	—	Articular cartilage	Apoptosis	↓
Yang et al., 2017	OA rat model	Treadmill exercise	LXA4	NF-κB	Articular cartilage	Inflammation	↓
Castrogiovanni et al., 2019	OA rat model	Physical Activity	IL-1β, IL-6, TNF-α, MMP-13	—	synovial	Inflammation	↓
Iijima et al., 2016	OA rat model	Treadmill walking	BMP-2, BMP-6	—	Subchondral bone	Cartilage matrix synthesis	↑
Boudenot et al., 2014	OA rat model	Interval training exercise			Subchondral bone	Apoptosis	↓
Iijima et al., 2017	OA rat model	Treadmill exercise	BMP-2, BMP-4, BMP-6, BMP receptor 2, pSmad-5	BMP	Articular cartilage, Subchondral bone	Inflammation, Apoptosis	↓
Oka et al., 2021	OA mice model	Treadmill exercise	Gremlin-1, MMP-13	—	Articular cartilage	Inflammation	↓
Vasilceac et al., 2021	OA rat model	Resistance training	MMP-2	—	Articular cartilage	Degradation of ECM	↓
Hong et al., 2014	OA rat model	Treadmill exercise	IL-6, EPAS-1, MMP-13	—	Articular cartilage	Degradation of ECM, Apoptosis	↓
Na et al., 2014	OA rat model	Treadmill exercise	C9	—	serum	Inflammation	↓
Cifuentes et al., 2010	OA rat model	Impact exercise	ACAN, COL2a1, TIMP3, MMP	—	Articular cartilage	Autophagy	↑
Yang et al., 2018	OA rat model	Treadmill exercise	LXA4	NF-κB	Synovial	Inflammation	↓
Hsieh and Yang, 2018	OA rat model	Swimming exercises	collagen II, MMP13	—	Articular cartilage, Synovial	Degradation of ECM, Apoptosis	↓
Hao et al., 2021	OA rat model	Body weight-supported treadmill training	collagen II, MMP13	—	Articular cartilage, Subchondral bone	Degradation of ECM, Apoptosis	↓
Iijima et al., 2015	OA rat model	Short-term gentle treadmill walking	—	—	subchondral bone	Apoptosis	↓
Al-Hashem et al., 2017	OA rat model	Swim exercise	TBARS, TNFa, SOD	—	Articular cartilage	Inflammation	↓
Martins et al., 2019	OA rat model	Aerobic training	MIA, IL1β, TNF	—	Articular cartilage	Inflammation	↓
Musumeci et al., 2015	OA rat model	Treadmill exercise	lubricin	—	Articular cartilage, Synovial	Cartilage matrix synthesis	↑
Musumeci et al., 2013	OA rat model	Treadmill exercise	caspase-3	—	Articular cartilage	Apoptosis	↓
Tian et al., 2021	OA rat model	Treadmill exercise	15-HETE	PI3k-Akt	Synovial	Inflammation	↓
Fallah Mohammadi et al., 2013	OA rat model	Treadmill exercise	IL – 1β, TNF – α	—	Articular cartilage	Inflammation	↓
Zhang J. et al., 2021	OA rat model	Swimming exercise	caspase-3	—	Articular cartilage	Apoptosis	↓
Li Z. et al., 2021	OA rat model	Treadmill exercise	P2 × 7	IRE1-mTOR-PERK	Articular cartilage	Autophagy	↑
Guan et al., 2011	OA chicken model	mechanical stimulation	HDAC4	—	Articular cartilage	miR-365	↑
Zhou et al., 2021	OA mice model	Treadmill running	—	—	Articular cartilage	lncRNA H19	↑
Liu Q. et al., 2016	OA cell model	mechanical stimulation	TMSB4	—	Articular cartilage	lncRNA-MSR	↑
Liu et al., 2017	OA cell model	mechanical stimulation	TNF-α	—	Articular cartilage	circRNA-MSR	↓
Fang et al., 2021	OA rabbit model	running experiment	PCNA, SOX9, Col II, Aggrecan	—	Articular cartilage	circUNK	↑

### The Role of Exercise in the Degradation of Extracellular Matrix

Exercise can delay the pathological process of OA by inhibiting the degradation of the ECM. Articular cartilage degradation is an essential pathological change of OA. Blazek et al. (2016) used OA rats as the research object. Exercise and non-exercise were used as intervention means to compare the whole gene expression of the transcriptome of articular cartilage in rats. Microarray analysis showed 644 differentially expressed genes in the articular cartilage of exercise rats. Therefore, exercise can prevent OA by changing genes related to OA’s pathogenesis and regulating the degradation and synthesis of ECM by changing metabolic pathways to alleviate the pathological changes of articular cartilage with sound prevention effects on OA. An OA rat model has been established to evaluate the expression of IL-10, TGF-β, and collagen type I and II in articular cartilage. The presentation of the above biochemical indicators increased in the articular cartilage of mice after exercise, indicating that exercise is very beneficial to articular cartilage. Moreover, considering that collagen type II is an essential component of ECM, exercise may delay the progression of OA by inhibiting the degradation of ECM and promoting cartilage synthesis (Assis et al., 2018). Vasilceac et al. (2021) conducted resistance training for eight weeks for OA rats. They analyzed whether ELISA changed the activity of MMP-2 in the tendons around the knee joint and finally found that exercise could reduce the activity of MMP-2 in the quadriceps tendon of OA rats and greatly relieve the adverse effects of OA on the knee joint. Results show that exercise may reduce the degradation of the ECM by inhibiting the activity of MMP-2 from achieving the purpose of treating OA. Exercise in different stages of the course of OA produces other effects. Type II collagen (CoII) and matrix metalloproteinase-13 (MMP-13) are essential components related to the degradation of the ECM. Hsieh and Yang (2018) established an OA rat model, in which the samples were allowed to swim in different stages of the course of OA. They found that swimming training in the early stage of OA could increase CoII. The level of MMP-13 can be reduced to maintain a balance between the degradation and anabolism of ECM and prevent cartilage damage through exercise training in the early stage of OA rather than that in the late stage.

Overall, the degradation of the ECM is the pathological change mechanism of OA. Exercise can protect articular cartilage and delay the progression of OA by inhibiting this mechanism, and it is a crucial intervention to prevent and treat OA.

### The Role of Exercise in Apoptosis

Apoptosis is one of the mechanisms for the pathological changes of OA. Exercise delays the pathological process of OA by inhibiting apoptosis. Taking articular cartilage of OA rats as a research sample, Yang et al. (2020) detected the expression of relevant genes in articular cartilage after exercise through bioinformatics analysis. Results show that the levels of TRAIL, NF-κB p65, and NLRP3 in cartilage after activity were decreased. Exercise could inhibit apoptosis and prevent OA by regulating the TRAIL/NF-κB/NLRP3 signaling pathway of OA. By comparing OA rats with mild, moderate, and high-intensity training and comparing the expression of caspase three and Hsp70, we found that mild and moderate exercise could inhibit apoptosis and protect the articular cartilage (Galois et al., 2004). Qian et al. (2014) established an OA rat model, and the experimental group was subjected to passive motion. Based on the measurements of the proteoglycan content of the cartilage matrix, the number of type II collagen fibers, and apoptotic chondrocytes in the experimental and control group, the changes in biochemical signals caused by passive exercise in the early and middle stages of OA differed. In comparison with the control group, the cartilage matrix proteoglycan content and type II collagen fiber level remarkably increased three weeks after passive motion in the early stage of OA. The number of apoptotic cells was significantly reduced. However, three weeks after passive exercise in the middle stage of OA, the number of apoptotic cells was not significantly changed. Therefore, passive exercise at the early stage of OA may delay articular cartilage degeneration by inhibiting apoptosis, preventing, and treating OA. After intermittent training for OA rats, the pathological changes of their subchondral bones were assessed by immunolabeling with cleaved caspase-3 in the cortical subchondral bone. Intermittent aerobic training may prevent the OA-induced reduction in bone mineral density by reducing apoptosis (Boudenot et al., 2014). Studies (Iijima et al., 2015) have established the OA rat model and analyzed the pathological conditions of rat articular cartilage and subchondral bone before and after four weeks of exercise. They found that exercise might inhibit apoptosis and protect cartilage tissue, thus delaying OA progression. Musumeci et al. (2013) established the OA rat model to study the effect of exercise on articular cartilage. First, drugs were used to induce pathological changes in the articular cartilage. Then, exercise intervention was performed on rats to observe the morphological changes of articular cartilage. The results show that exercise might induce the expression of lubricating oil and inhibit the activity of caspase-3, thereby reducing apoptosis and delaying or even reversing pathological changes of articular cartilage. In some studies (Zhang J. et al., 2021) concerning establishing the OA rat model, the experimental group was intervened by swimming for four weeks. The morphological changes of cartilage were analyzed by hematoxylin-eosin (H&E) staining. The expression of caspase-3 was analyzed by Western blot and qPCR. The results showed that after four weeks of swimming, the abnormal morphology of articular cartilage was improved, and the level of caspase-3 protein decreased, indicating that exercise might reduce apoptosis by inhibiting the level of caspase-3 protein, thereby protecting articular cartilage and preventing and treating OA.

### The Role of Exercise in Inflammatory Response

Exercise can inhibit the inflammatory response by reducing pro-inflammatory factors, thereby delaying the pathological changes of OA, which is the most common mechanism of activity in OA treatment. The inflammatory response mechanism of exercise therapy for OA has been widely studied, and many related pathways regulate the inflammatory response of OA, such as PI3k/Akt, NF-κB p65, JNK/NF-kB, HDAC3/NF-kappaB, and AMPK/NF-κB signaling pathways. Lu et al. (2021) established the OA rat model and allowed them to exercise on the treadmill. They found that after running exercise, the mice produced maresin-1, and the increased level of maresin-1 activated the PI3k/Akt pathway and inhibited the NF-κB p65 pathway, thus playing an anti-inflammatory role and delaying the pathological changes of OA. Griffin et al. (2012) established the OA mouse model by inducing a high-fat diet and then letting the mice run to observe the expression of inflammation-related factors. Finally, they found that exercise could regulate the expression of pro-inflammatory factors, promote joint health, and reduce the severity of pathological changes in joints of OA mice. To study the signal transduction of JNK/NF-kB in KOA patients, we established the 0A rat model and conducted a controlled intervention experiment. Finally, the knee joint diameter in the exercise group is lower than that in the OA group, indicating that exercise is conducive to KOA recovery. The IL-1b, IL-6, and TNF-a levels decreased, indicating that exercise can reduce the inflammatory response by regulating the JNK/NF-kB signaling pathway, which ultimately can delay the pathological changes of OA (Chen et al., 2020). Osteoarthritis rats were subjected to moderate-intensity exercise on the treadmill. The H&E staining and toluidine blue O staining were used to detect cartilage injury. The expression levels of some biochemical signals in the articular cartilage were examined via immunohistochemistry and other methods. The results showed that moderate-intensity treadmill exercise could reduce the inflammatory response and protect the articular cartilage by inhibiting the HDAC3/NF-kappaB pathway (Zhang H. et al., 2019). Similarly, Yang et al. (2019) intervened OA rats with treadmill exercise and used the articular cartilage as the research object. Observation and analysis results show that moderate-intensity exercise can reduce the sensitivity of articular cartilage and chondrocytes to inflammatory response through the AMPK/NF-κB signaling pathway, and it is an essential mechanism for reversing the pathological changes of articular cartilage. The analysis of biochemical indicators of OA rats after aerobic exercise show that the expression levels of IL-1β, caspase-3, and MMP-13 in OA rats decreased after exercise, and aerobic exercise could inhibit the inflammatory response of OA and prevent and treat the pathological changes of cartilage (Assis et al., 2016). After water sports, OA rats’ articular cartilage and inflammatory mediators were changed. After eight weeks of the experiment, the cell damage degree of the rat was relieved, and the expression levels of IL-1β and caspase-3 were reduced. Therefore, sports can regulate OA inflammatory factors and inhibit inflammatory reactions to treat OA (Milares et al., 2016).

### The Role of Exercise in Autophagy

In OA, autophagy is a cellular protective response. Exercise can protect the knee joints of KOA patients by inducing autophagy, which is mainly regulated by oxidative stress. Relatively few studies have demonstrated the specific mechanism pathways through which exercise protects the knee joint through autophagy. Zhang X. et al. (2019) used sodium iodoacetate to induce OA rat model, and the experimental group was subjected to treadmill exercise for four weeks. The study found that serum IL-1β was decreased, IL-4 was increased, and the expression of type II collagen in articular cartilage was increased in rats undergoing treadmill exercise compared with those in OA rats that did not experience any activity. Therefore, treadmill exercise may protect the knee joints of OA patients by promoting the autophagy of articular cartilage. Baur et al. (2011) established an OA mouse model to study the effect of running on articular cartilage and bone of OA patients and observed the changes of biochemical signals and articular cartilage of running mice after eight weeks. They found that the content of reactive oxygen species in mice increased after exercise. Exercise can promote autophagy and protect the articular cartilage and bone of OA patients by regulating oxidative stress. Another study (Cifuentes et al., 2010) has established an OA rat model. The experimental group was exposed to treadmill exercise for 8 weeks to observe the pathological changes of articular cartilage. In comparison with OA rats that did not undergo exercise, the protein and polysaccharide contents on the surface of the articular cartilage of rats after eight weeks of the exercise was relatively high. This experiment confirmed that training can enhance the oxidative stress mechanism and protect articular cartilage. Moderate-intensity exercise plays different roles in different stages of OA progression. In the early stage of OA, the average power of movement can delay the progression of OA by promoting autophagy. In the late stage of OA, moderate-intensity exercise can over-activate purinergic receptor P2X ligand-gated ion channel 7 and increase the number of apoptotic cells, thus aggravating the progression of OA. Throughout the OA progression, the body may promote autophagy or apoptosis through the IRE1-mTOR-PERK signal axis to delay or accelerate the pathological process of OA (Li Z. et al., 2021).

### The Role of Exercise in Non-coding RNA

Exercise can alleviate the pathological changes of OA by regulating the expression of ncRNA. The practice affect the face of miRNA, lncRNA, and circRNA in articular cartilage, synovial membrane, and subchondral bone of OA patients.

miRNA is related to maintaining the typical morphology of articular cartilage. The detection of miRNA expression in OA rats revealed that compared with the non-exercise rats, 394 differentially expressed miRNAs were detected in the exercise rats (Yang et al., 2020). The analysis of articular cartilage from different animals revealed that exercise could change the miRNA expression and improve the pathological changes of articular cartilage through a series of mechanisms. Dunn et al. (2009) analyzed the expression of miRNA in bovine articular cartilage. They found that the expression patterns of miR-221 and miR-222 in weight-bearing medial anterior condylar articular cartilage was higher than those in non-weight-bearing medial posterior condylar articular cartilage. When the articular cartilage was loaded with weight, miR-221 downregulated type II collagen and Sox9, and miR-222 downregulated HDAC4 and MMP-13, maintaining the typical morphology of articular cartilage. Guan et al. (2011) subjected chicken chondrocyte samples to mechanical stimulation to analyze the roles of relevant miRNA in the mechanical stimulation using microarray technology. Results show that mechanical stimulation could upregulate the miR-365 expression in chondrocytes. miR-365 stimulated the differentiation of chondrocytes by targeting histone deacetylase 4 (HDAC4) and finally alleviated the pathological process of cartilage tissue. In addition, the expression levels of many lncRNA changed during exercise. Zhou et al. (2021) established an OA mouse model. Mice with moderate-intensity exercise intervention had an upregulated lncRNA H19 expression, thickened articular cartilage, and resolved OA compared with mice without exercise intervention. Under the stimulation of mechanical stress, lncRNA-MSR was activated and then competitively bound to miR-152, thereby promoting the expression of TMSB4 and leading to the degradation of the ECM (Liu Q. et al., 2016). The regulatory role of circRNA during exercise is also crucial. A total of 104 circRNA were differentially expressed (44 upregulated and 60 downregulated), with increased circRNA-MSR expression, in damaged articular cartilage compared with intact articular cartilage. circRNA-MSR expression was downregulated after mechanical stress was applied to the cartilage. The downregulation of circRNA-MSR can inhibit the expression of TNF-α and promote the degradation of the extracellular chondrocyte matrix, thus enhancing the structure and function of cartilage (Liu et al., 2017). In addition, circRNA-MSR can directly target miR-643 to u-regulate MAP2K6. The downregulation of circRNA-MSR expression can promote cell proliferation and reduce cartilage damage (Jia and Wei, 2021). qRT-PCR and Western blot have been used to analyze the genes related to cartilage differentiation in cartilage tissues. The results show that the expression of circUNK was upregulated, which improved the cartilage injury in OA rabbit and promoted the expression of molecules related to cartilage differentiation, such as PCNA, SOX9, Col II, Aggrecan, and protein-polysaccharide. These molecules had sound effects on the proliferation and differentiation of articular cartilage. In conclusion, exercise or mechanical stimulation can lead to changes in the expression of ncRNA, which prevents and improves the pathological changes of articular cartilage, synovial membrane, and subchondral bone through a series of mechanisms, thereby delaying the progression of OA (Fang et al., 2021).

## Effects of Different Exercise Types on Human Osteoarthritis

Many types of training can relieve pain, enhance muscle strength and improve joint stiffness (Daenen et al., 2015). For healthy people, exercise can promote good health, while for OA patients, dysfunction can be improved and diseases can be treated (Skou et al., 2018). At present, there are many types of sports for the treatment of OA, such as aerobic exercise, anti-resistance exercise, neuromuscular training, etc (Jansen et al., 2011). In addition, Chinese traditional sports such as Baduanjin, Wuqinxi, and yoga are also applied to the prevention and treatment of OA (Li et al., 2020). This paper summarizes the research on training types of OA patients by summarizing related literature, as shown in Table 2.

**TABLE 2:** Different exercise types on human Osteoarthritis (OA).

Researchers	Number of studies/subjects	Intervention studied	Exercise effect
Vincent et al., 2019	*N* = 90	Eccentric and Concentric Resistance Exercise	Modify function and pain symptoms
DeVita et al., 2018	*N* = 30	Quadriceps strength training	Increase muscle strength and improve symptomatic and functional outcomes
Abbott et al., 2013	*N* = 206	Manual therapy and exercise therapy	Improve knee joint function of patients
Lund et al., 2008	*N* = 79	A land-based exercise	Improve pain and muscle strength
Chen S.-M. et al., 2019	*N* = 70	Resistance Exercise	Improve muscle strength, dynamic balance and body function
Kabiri et al., 2018	*N* = 78	Aerobic exercise combined with resistance training	Relieve pain and improve function
Allen et al., 2021	*N* = 345	Stepped exercise	Improve the symptoms of the patient
Bennell et al., 2014b	*N* = 100	Neuromuscular versus quadriceps strengthening exercise	Relieve pain and improve function
Ettinger et al., 1997	*N* = 439	An aerobic or a resistance exercise	Relieve pain and improve function
Suzuki et al., 2019	*N* = 52	Multiple exercise	Improve muscle strength and joint flexibility
Krauß et al., 2014	*N* = 209	Exercise Therapy	Relieve pain and improve function
León-Ballesteros et al., 2020	*N* = 32	Quadriceps strengthening exercise	Relieve pain
Rewald et al., 2020	*N* = 55	Aquatic Cycling	Relieve pain and improve function
Chen H. et al., 2019	*N* = 171	A home-based exercise	Relieve symptoms, increase the physical functioning, and improve quality of life
Alkatan et al., 2016	*N* = 48	Swimming and Cycling Training	Reduce joint pain and stiffness and improve muscle strength and functional capacity
Silva et al., 2008	*N* = 64	Water-based and land-based exercises	Relieve pain and improve function
Kuntz et al., 2018	*N* = 31	Yoga	Relieve pain and improve function
Huang et al., 2018	*N* = 250	Quadriceps functional exercise	Relieve pain and improve function
Taglietti et al., 2018	*N* = 31	Aquatic exercises	Relieve pain and improve function
Vincent and Vincent, 2020	*N* = 88	Resistance exercise	Relieve pain
Holm et al., 2021	*N* = 90	Strength training	Relieve pain
Waller et al., 2017	*N* = 87	High intensity aquatic resistance training	Decrease fat mass and improve walking speed
Fernandes et al., 2017	*N* = 165	Neuromuscular exercise	Improve the quality of life of patients
Ferraz et al., 2018	*N* = 48	Resistance Training	Relieve pain and increase muscle strength
Hsu et al., 2021	*N* = 66	Resistance Training	The body composition, blood biochemistry and lower limb function of patients were improved.
Devrimsel et al., 2019	*N* = 60	Neuromuscular electrical stimulation	Relieve pain and improve function
Hinman et al., 2007	*N* = 71	Aquatic physical therapy	Relieve pain and improve physical function, strength, and quality of life
Xiao et al., 2021	*N* = 68	A Wuqinxi exercise	Relieve pain and improve function
Yuenyongviwat et al., 2020	*N* = 86	Hip abductor strengthening exercises	Expedite improvement of less pain, symptoms, activity in daily living and quality of life
Rabe et al., 2018	*N* = 42	The Combined Application of Neuromuscular Electrical Stimulation and Volitional Contractions	Alleviate pain and improve physical performance
Lai et al., 2018	*N* = 40	Strength exercise	Improve the knee flexion proprioception
Steinhilber et al., 2017	*N* = 210	Tübingen exercise therapy	Increase muscle strength
Kuptniratsaikul et al., 2019	*N* = 80	Underwater treadmill exercise	Relieve pain and improve function
Oliveira et al., 2012	*N* = 100	Quadriceps strengthening exercises	Improve pain, function, and stiffness
Wang et al., 2007	*N* = 38	Aquatic exercise	Improve knee and hip flexibility, strength and aerobic fitness
Nery et al., 2021	*N* = 60	A progressive resistance exercise	Relieve pain and improve function
Wang et al., 2016	*N* = 39	Whole-body vibration training with quadriceps strengthening exercise	Improve symptoms, physical function and spatiotemporal parameters
Pisters et al., 2010	*N* = 200	Behavioral graded activity	Increase the patient’ s exercise compliance and physical activity
An et al., 2013	*N* = 28	Baduanjin	Relieve pain and improve function
Trans et al., 2009	*N* = 52	Whole body vibration	Increase muscle strength and proprioception
Sevick et al., 2000	*N* = 439	Resistance training	Improve physical function
Ojoawo et al., 2016	*N* = 45	Proprioceptive exercises	Relieve pain and joint stiffness
Villadsen et al., 2014	*N* = 165	Neuromuscular exercise	Relieve pain and improve activity of daily living
Wang et al., 2021	*N* = 128	Quadriceps strengthening exercises	Relieve pain, increase physical function, improve self-efficacy and improve quality of life.
Jorge et al., 2015	*N* = 29	Progressive resistance exercise	Relieve pain, improve function and improve quality of life.
Penninx et al., 2002	*N* = 439	Aerobic and resistance exercise	Relieve pain and improve walking speed.
Simão et al., 2012	*N* = 32	Whole-body vibration training	Improve patients’ self-perception of pain, balance, gait quality and inflammatory markers.
Lun et al., 2015	*N* = 72	Hip and leg strengthening exercise	Improve pain, function, and quality of life
Maurer et al., 1999	*N* = 113	Isokinetic quadriceps exercise	Relieve pain and improve function
Bruce-Brand et al., 2012	*N* = 41	Resistance training and neuromuscular electrical stimulation	Improve function, disability and pain
Hiyama et al., 2012	*N* = 40	Walking exercise	Improve executive function and dual-task performance
Foley et al., 2003	*N* = 105	A hydrotherapy resistance exercise with a gym based resistance exercise	Improve muscle strength and function.
Tsauo et al., 2008	*N* = 60	Sensorimotor training	Improve the patients’ proprioception in the knee joints and their self-reported function
Topp et al., 2002	*N* = 102	Dynamic or isometric resistance training	Improve functional ability and reduce knee joint pain
Munukka et al., 2016	*N* = 87	Aquatic resistance training	Improve cardiorespiratory fitness
Samut et al., 2015	*N* = 42	Isokinetic and aerobic exercise training	Enhance muscle strength and functional ability
Wang et al., 2011	*N* = 84	Aquatic exercises and land-based exercises	Relieve pain, improve knee joint range of motion and quality of life
Chen P.-Y. et al., 2021	*N* = 68	Tai chi exercise	Enhance physical function
Song et al., 2003	*N* = 72	Tai chi exercise	Improve symptoms, balance, and physical functioning.

## Conclusion and Outlook

At present, the incidence of OA is very high, and its pathogenesis remains unclear. The treatment of OA by exercise has received increasing research attention. Not all types of sports used can alleviate OA. The result of different types, intensity, and time of sports on OA differs.

Some studies believe that exercise does not affect OA. For example, Rios et al. (2020) intervened in KOA rats with aerobic exercise for 12 weeks and measured bone mineral density and knee joint injury. The results showed that training did not affect the common knee injury, accelerate the progression of OA, and alleviate the pathological changes of OA. Other studies believe that exercise accelerates the progression of OA. For example, Liu S.-S. et al. (2016) established a rat model of exercise-induced OA. They analyzed and compared the expression levels of various related mRNA, protein, and pathways in the standard and injury group of exercise-induced OA by using RT-qPCR, Western blot analysis, and immunohistochemical staining. The results showed that excessive pressure would lead to abnormal expression of many biochemical signals and abnormal activation of the Wnt/β-catenin pathway, leading to induced OA. Exercise has different effects on healthy and damaged articular cartilage (Rojas-Ortega et al., 2015). Notably, for articular cartilage injury, if high-intensity practice is continued, the activity of catabolic proteins is more vital than that of anabolic proteins. The body’s metabolism is imbalanced, thus efficiently inducing OA. Twelve weeks of high-intensity treadmill training in rats will increase the levels of some apoptosis-related proteins and inflammation-related factors, such as caspase-3, IL-1α, and TNF-α, which is the precursor of OA (Franciozi et al., 2013). The results indicate that high-intensity exercise may lead to the occurrence and development of OA. In addition, moderate exercise can delay the pathological process of OA. For example, a daily external force has been used to compress OA mice under low or medium intensity, and the pathological changes of articular cartilage were observed in the second and sixth weeks. Moreover, low- and medium-intensity compression can reduce the degeneration of articular cartilage and osteophytes of subchondral bone in mice and is very beneficial to the damaged joints, making it an effective method for the treatment of OA (Holyoak et al., 2019).

At present, many exercises have therapeutic effects on OA, such as aerobic exercise, strength training, swimming, neuromuscular exercise, proprioceptive training, and balance training. Different types of motion produce other effects. Aerobic exercise is the most widely used in OA patients, and it can reduce the expression of IL-1β, caspase-3 and MMP-13 and prevent cartilage degradation in KOA rats (Assis et al., 2016). Aerobic exercise may be the best training method to reduce pain and improve body function (Goh et al., 2019). However, aerobic exercise with different intensities has different effects on OA patients with varying degrees of injury. Low-intensity aerobic exercise had a better therapeutic effect in patients with severe OA (Messier et al., 2021), while high-intensity aerobic exercise had a better therapeutic effect in patients with mild OA (Multanen et al., 2017). In addition, strength training could reduce the activity of MMP-2 in the quadriceps tendon of the OA rat model (Vasilceac et al., 2021), which was the most effective in improving muscle strength, and neuromuscular training was the best training method to relieve OA pain (Ageberg and Roos, 2015). Swimming intervention in the early stage of OA can alleviate the stiffness of the knee joint and is better than land exercise (Lund et al., 2008; Hsieh and Yang, 2018; Munukka et al., 2020). Land exercise can be performed after the patients’ joints have a certain degree of flexibility. Land exercise is better than water exercise in improving pain and enhancing function (Escalante et al., 2011). Many traditional techniques are being applied more and more in OA, such as Baduanjin (An et al., 2008), tai chi chuan (Zhang Z. et al., 2020), Wuqinxi (Xiao and Li, 2021), and yoga (Kuntz et al., 2018). They can improve the physical function of OA patients and have a significant impact on the psychological status of OA patients. In addition, new intervention methods such as virtual reality and sports games have been gradually used to improve people’s physical and psychological conditions (Sadeghi and Jehu, 2022). These sports have excellent development prospects in the treatment of OA.

Although many types of exercise can be used to prevent or treat OA, our most recommended method is to formulate a unique exercise prescription for each patient with OA based on the FITT (Frequency, Intensity, Time, and Type) principle. According to the exercise prescription, a study (de Rooij et al., 2017) formulated a personalized rehabilitation program for 126 OA patients for 20 weeks. It was finally found that personalized exercise therapy could effectively improve the OA of the knee joint and the body function of the patients. Exercise prescription recommendations for patients with OA are as follows (Bennell et al., 2014a): (1) Exercise frequency: It is recommended that in the initial stage, the patient had better exercise at least 12 times within three months to master the skills and ensure compliance, and then the frequency was gradually increased and maintained to two to three times a week, and a week of moderate-intensity exercise lasting 150 to 300 min was accumulated; (2) Exercise intensity: Moderate intensity exercise is recommended. Namely, the heart rate is 120–150 beats/min, and the oxygen consumption during exercise is 50–70% of the maximum oxygen consumption; (3) Exercise time: The recommended exercise time is 30–60 min per day. The exercise time was gradually increased from a short time to 30–60 min/d; (4) Exercise type: generally, aerobic exercise and strength training are recommended. Aerobic exercise such as swimming, Tai Ji Chuan, unfavorable for mountaineering, climbing stairs and other excessive weight-bearing movements, strength training have equal length, such as Zhang or isokinetic resistance movement, etc., recommend the weight-bearing exercise is given priority to; (5) Precautions: Pain relief can be obtained only after 8–11 weeks of exercise therapy. Continuous exercise for more than 12 weeks is generally recommended to relieve decreased muscle strength and muscle atrophy associated with OA. Patients should also receive regular education during training to improve their self-management awareness, compliance and exercise efficacy. It is normal to feel some discomfort or pain during exercise. Ice application is required at the joints for 15–20 min after training. If severe pain occurs during exercise or swelling aggravates the next day, you should timely adjust the amount of activity. Different types of patients may have other exercise prescriptions. Patients with higher obesity are more suitable for aquatic exercise. For patients with upper limb OA, emphasis should be placed on improving the affected joints’ range of motion and flexibility. For patients with lower limb OA, emphasis should be placed on improving patients’ muscle strength and body stability. Therefore, we should formulate individualized exercise prescriptions according to the degree of lesion and needs of OA patients.

This review describes the pathological change mechanism of OA and the molecular mechanism of exercise in the treatment of OA. By using RT-qPCR, Western blot analysis, and immunohistochemical staining, we have found that the expression levels of related mRNA, protein, and pathways were changed in OA patients after exercise. This review also summarized animal experiments related to exercise and OA and the role of different exercise types in human OA, described the research progress of exercise in the prevention and treatment of OA, and provided a basis for exercise intervention to treat OA in the future. Exercise, as an intervention means, has a great development prospect in the treatment of OA.

## Author Contributions

X-AZ and X-QW: conceptualization, project administration, and funding acquisition. HK, X-AZ, and X-QW: writing – review and editing. All authors contributed to the article and approved the submitted version.

## Conflict of Interest

The authors declare that the research was conducted in the absence of any commercial or financial relationships that could be construed as a potential conflict of interest.

## Publisher’s Note

All claims expressed in this article are solely those of the authors and do not necessarily represent those of their affiliated organizations, or those of the publisher, the editors and the reviewers. Any product that may be evaluated in this article, or claim that may be made by its manufacturer, is not guaranteed or endorsed by the publisher.
